# Innovative novel regularized memory graph attention capsule network for financial fraud detection

**DOI:** 10.1371/journal.pone.0317893

**Published:** 2025-05-28

**Authors:** Xiangting Shi, Xiaochen Wang, Yakang Zhang, Xiaoyi Zhang, Manning Yu, Lihao Zhang

**Affiliations:** 1 Industrial Engineering and Operations Research Department, Columbia University, New York, New York, United States of America; 2 Department of Finance, The London School of Economics and Political Science, London, United Kingdom; 3 College of Liberal Arts and Science, University of Illinois Urbana-Champaign, Illinois, United States of America; 4 Department of Statistics, Columbia University, New York, New York, United States of America; 5 Department of Information Engineering, The Chinese University of Hong Kong, Shatin, N.T., Hong Kong; Tata Consultancy Services Ltd., UNITED STATES OF AMERICA

## Abstract

Financial fraud detection (FFD) is crucial for ensuring the safety and efficiency of financial transactions. This article presents the Regularised Memory Graph Attention Capsule Network (RMGACNet), an original architecture aiming at improving fraud detection using Bidirectional Long Short-Term Memory (BiLSTM) networks combined with advanced feature extraction and classification algorithms. The model is tested on two reliable datasets: the European Cardholder (ECH) transactions dataset, which contains 284,807 transactions and 492 fraud instances, and the IEEE-CIS dataset, which has more than 1 million transactions. Our approach enhances comparison to existing methods of feature selection and classification accuracy. On the ECH dataset, RMGACNet achieves an accuracy of 0.9772, a precision of 0.9768, and an F1 score of 0.9770 measures; on the IEEE-CIS dataset, it achieves an accuracy of 0.9882, a precision of 0.9876 and an F1 score of 0.9879. The findings indicate that RMGACNet routinely surpasses existing models’ efficiency and accuracy while ensuring strong execution time performance, especially when handling large-scale datasets. The suggested model demonstrates scalability and stability, making it suitable for real-time financial systems.

## Introduction

Through illegal activities, financial fraud compromises banks, lending institutions, payment systems, and fintech platforms [1]. Credit card fraud is especially prominent among financial crimes as it involves illegally acquiring significant quantities of money without the cardholder’s permission. As such, financial institutions must spot and stop fraudsters copying transaction methods. Big data analytics and artificial intelligence (AI) have drastically changed mobile transactions in the last 10 years and monetary transfers via digital wallets. Criminals have improved their strategies, learning to cash credit and counterfeit cards to exploit flaws in technologically driven payment systems. Recent artificial intelligence discoveries like attention processes and graph neural networks (GNNs) have generated new chances for fraud detection. Some approaches, like memory-augmented neural networks and capsule networks (CapsNet), have shown promise in helping fraud detection systems deal with class imbalance and malicious attacks [2]. These networks maintain hierarchical links among items. According to Graph Attention Networks (GATs), the topological properties of transaction data have been used to enhance fraud detection [3]. Recent advances in deep learning have given banks powerful new weapons in the fight against fraud.

Complex market fraud affects consumers and economies worldwide. Professionals continuously seek innovative ways to fight fraud, including credit card theft. The use of data mining and machine learning has dramatically improved financial fraud prevention [4]. Machine learning algorithms search big datasets for fraudulent transaction patterns, lowering susceptibility. These algorithms struggle with credit card fraud datasets because illegal transactions frequently lead to genuine ones. Since financial fraud is rising in the digital era, fraud detection systems must be flexible. Understanding the ever-changing credit card fraud landscape is crucial for effective defences [5]. Illicit operations, money laundering, and identity theft make the banking sector prone to fraud. Fraud like this may waste cash and damage business confidence. Despite security improvements, scammers adapt their methods [6, 7]. Thus, proactive and flexible detection systems are essential nowadays. Despite their utility, rule-based techniques cannot handle the ever-changing fraud scenario. AI and ML help prevent fraud by detecting anomalous transactions and subtle patterns in massive data sets. In the future, machine learning algorithms may discover new fraud and red flags that older approaches miss. Despite its fraud detection potential, machine learning faces several challenges. A significant distinction is between real and fake credit card transactions. As a result, algorithms can benefit real-world transactions above fraud detection, reducing their overall efficiency [8]. Resampling, cost-sensitive learning, and anomaly detection handle these constraints. Academics and practitioners aspire to design fraud detection systems with fewer false positives and higher accuracy utilizing these methods. Fraud detection systems must also tackle adversarial attacks—criminals utilize AI algorithms to make untraceable transactions. The growing conflict between fraudsters and anti-fraud systems requires scientists and practitioners to collaborate [9].

Regularized Memory Graph Attention Capsule Networks increase fraud detection. Using a unique technique, memory systems, graph attention processes, and capsule networks identify authentic data from fake financial transactions. The research uses half a million records and 350 characteristics. Therefore, plan properly to prevent embarrassing yourself. The Synthetic Minority Oversampling Technique (SMOTE) corrects dataset class imbalances, a significant fraud detection problem. After data balancing, feature engineering and selection preserve the most essential qualities. This strategy improves model training and minimizes computing complexity. RMGACNet blends deep learning and graph-based representation learning to detect fraud. This comprehensive technique enhances accuracy and reduces false positives while adapting to new fraud tendencies. Key points of this article:

1. An enhanced deep learning approach named RMGACNet combines Regularized Memory Graph Attention and CapsuleNet architecture, which improves detection by utilizing temporal and contextual data.
2. An upgraded Memory Graph Attention Mechanism prioritizes critical transactional data in RMGACNet. This helps the system identify complicated patterns and anomalies that indicate fraud.
3. The approach reduces overfitting with targeted regularisation to increase model generalizability. This method makes RMGACNet efficient in many real-world circumstances and datasets, making it suitable for fraud detection applications.
4. Real-world data has been utilized extensively to empirically validate the ECH and IEEE-CIS datasets. RMGACNet outperforms state-of-the-art approaches and published studies in detecting fraudulent transactions with a low false positive rate, making it helpful for real-world fraud detection.
5. Scalability and Computing Efficiency: RMGACNet balances computational economy and model fidelity to minimize execution times compared to older methods. This efficiency allows real-time fraud detection systems to respond quickly and reduce financial losses.
6. Examining space sensitivity, memory utilization, and computational complexity illustrates RMGACNet’s resource demands. This research assesses this approach’s usefulness and scalability in financial fraud detection.

Furthermore, RMGACNet’s enhanced performance, efficiency, and flexibility made financial fraud detection a breakthrough. The following sequence is followed for the rest of the subsequent sections: The Related Work section discusses the relevant research, followed by the Proposed System Model section, which presents the proposed model. The Simulation and Results Discussion section provides simulation findings, and finally, the Conclusion section summarizes the paper.

## Related work

Credit cards have attracted considerable attention since their introduction owing to their incorporation of online banking and consumer electronics. Due to their significant relevance, many experts have meticulously analyzed the topic of credit card theft in recent years. Traditionally, credit card fraud detection was conducted using standard machine learning approaches, such as Support Vector Machines (SVM) and Markov models, prior to the advent of deep learning methodologies.

One study used a technique for detecting fraud using Support Vector Machines (SVMs) and evolutionary algorithms, which was suggested in [10] for use with constrained sample data that deviates from a normal distribution. The Hidden Markov Model (HMM) was used in [11] to optimize the process and enhance the Identification of fraudulent transactions. A bagging ensemble classifier based on a decision tree method was presented in [12]. This strategy addresses real-time reasoning and the disparity in credit card transaction data categories by applying it to a genuine credit card transaction dataset. A comparative analysis of Bayesian classifiers for credit card fraud detection was conducted in [13], revealing that all classifiers within the PCA dataset exhibited enhanced detection accuracy. Given the complexities of real-world debit card use, while machine learning may mitigate financial fraud, further research is required to rapidly and accurately discern prevalent characteristics from constrained transaction data.

Fraud detection using a convolutional neural network was proposed in [14]. Labelled data helps this network discover fraud tendencies and determine transaction sample fraud. In mobile communication network experiments, Deep Convolutional Neural Networks (DCNNs) outperformed other machine learning algorithms [15]. Nonetheless, the approaches encounter significant hurdles owing to data asymmetry in financial fraud datasets.

The [16] author recommended investigating fraud detection and analyzing credit card data variability in the frequency domain for more accurate and consistent data representation. To mitigate class imbalance in financial fraud transaction data and improve classifier efficacy, the authors of [17] used Generative Adversarial Networks (GAN) to provide samples for the minority class. In [18], the researcher examined Fourier and Wavelet transformations in proactive fraud detection. Furthermore, researchers in [19] devised a credit card fraud detection system by amalgamating LSTM and AdaBoost algorithms, enhancing performance using data resampling techniques inside the learning process.

To improve the precision, accuracy, and outcomes of machine learning processes for unbalanced data, the authors of [20] used an under-sampling technique. They promoted integrating datasets and using the fuzzy C-means method to discern standard drawings defined by data integrity attributes and notable fraud. proposed three innovative additions [21], including a Deep Stacking Autoencoder (DSA) that is data-balanced using a Harris Grey Wolf (HGW) methodology. This study advocates for the training of the DSA using the HGW network, which has shown superior efficacy in fraud detection according to fitness measures. This fitness technique continuously attains optimal results by exercising with few mistakes. The authors of [22] used many machine learning models, including decision trees, Naive Bayes, logistic regression, XGBoost, and random forest, to identify credit card fraud. A fully connected neural network system using class oversampling was developed to detect misbehaviour [23]. This research confirmed class distribution using biassed target data, highlighting the need for sophisticated detection techniques in the changing realm of credit card fraud. Table 1 describes the summarized view of the literature review.

**Table 1:** Overview of current literature in FFD.

Ref	Problem Identified	Method Used	Objective Achieved	Limitations
[10]	Detection of credit card fraud with few-sample data that is not normally distributed	SVM and evolutionary algorithms	Simplifying fraud detection and Identification of fraudulent transactions	Limited applicability to sparse data
[11]	Need for simplification in identifying fraudulent transactions	Hidden Markov Model (HMM)	Simplification and improved identification of fraudulent transactions	Limited scalability to complex fraud patterns
[12]	Real-time reasoning and addressing category imbalance in credit card transaction data	Decision Tree algorithm-based bagging ensemble classifier	Real-time reasoning and addressing category imbalance	Dependency on specific algorithm and dataset
[13]	Comparison of Bayesian classifiers’ efficacy in detecting credit card fraud	Bayesian classifiers	High detection accuracy for all classifiers	Limited exploration of other ML techniques
[14]	Learning inherent patterns of fraudulent behavior from labeled data	Convolutional Neural Network (CNN)	Improved fraud detection based on learned patterns	Limited scalability to complex fraud patterns
[15]	Investigation of Deep CNN (DCNNs) in fraud detection	DCNNs	Superior performance in fraud detection, particularly in mobile communication networks	Limited exploration of other DL techniques
[16]	Obtaining consistent data representation for fraud detection	Examination of the variety of credit card data in the frequency domain	Improved data representation for fraud detection	Limited applicability to specific data representations
[17]	Addressing class imbalance in financial fraud transaction data	Generative Adversarial Networks (GANs)	Improved classifier performance through minority class sample generation	Dependency on GAN performance
[18]	Investigating proactive fraud detection using Fourier and Wavelet transforms	Fourier and Wavelet transforms	Improved proactive fraud detection	Limited exploration of other proactive detection techniques
[19]	Efficient credit card fraud detection with data resampling and integrated learning methodologies.	Long Short-Term Memory (LSTM) and AdaBoost	Robust fraud detection through integrated learning	Dependency on dataset quality and class distribution
[20]	Processing imbalanced data for improved accuracy and precision	Under-sampling approach	Improved accuracy, precision, and outcomes	Limited exploration of other data balancing techniques
[21]	Development of a data-balanced DDSA for fraud detection	Deep Stacking Autoencoder (DSA) based on the Harris Grey Wolf (HGW) network	Improved fraud detection through enhanced feature extraction	Dependency on network architecture and training process
[22]	Identification of fraudulent charges on credit cards using various ML models	XGBoost, Logistic Regression, Naive Bayes, Random Forest, decision trees,	Detection of fraudulent charges on credit cards	Limited exploration of DL techniques
[23]	Fraud detection with class oversampling and a fully linked neural network architecture	Fully linked neural network with class oversampling.	Improved fraud detection through oversampling and deep learning	Dependency on dataset quality and class distribution

## Proposed system model

Our framework adheres to a sequence of systematically organized phases, beginning with dataset preparation and culminating in model evaluation and training. The raw input data, including 2023 European cardholder transactions, is preprocessed to guarantee cleanliness and uniformity of attributes. This includes imputing missing values, removing outliers, normalizing and standardizing data, encoding labels, and scaling features. The Synthetic Minority Over-sampling Technique (SMOTE) equalizes fraudulent and non-fraudulent instances in fraud detection datasets to correct class imbalance. SMOTE was chosen because it improved model performance by providing synthetic minority class data points. Feature selection uses correlation analysis and feature engineering to reduce noise and increase model performance to find the most essential attributes. Using domain expertise and correlation analysis, feature engineering developed significant features that increase model accuracy by better-describing data. RMGACNet uses advanced methods such as BiLSTM, GraphSAGE, Capsule Networks, Attention Mechanisms, Graph Attention Networks, and L2 Regularization. These layers were selected for their various fraud detection benefits:

- The temporal associations in the transaction data were captured using BiLSTM.
- The graph-based learning techniques of GraphSAGE and Graph Attention Networks (GAT) were used to uncover fraud patterns and clarify the nature of the relationships between transactions.
- Capsule networks are useful for detecting complex fraud patterns because they allow for modelling spatial hierarchies and maintaining spatial links.
- By enhancing the model’s focus on important components, attention approaches ensure that critical fraudulent indications are not neglected.
- Grid search and cross-validation systematically optimized hyperparameters to enhance performance, including the number of layers, learning rate, and regularization parameters.
- L2 regularization mitigated overfitting, ensuring a balance between model complexity and generalization.

**Fig 1:**
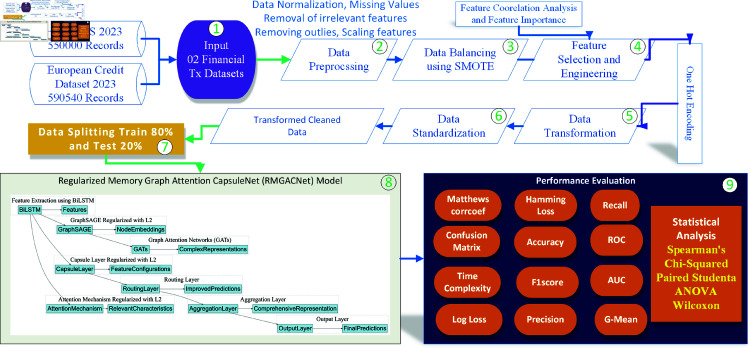
Proposed framework for FFD.

After training and testing on the balanced and preprocessed dataset, the model is evaluated for its ability to discern fraud from honest financial transactions, demonstrating its effectiveness in real-world applications. The whole architecture is shown in Fig 1, and the pseudocode for the proposed model’s algorithm is outlined in Algorithm 1.

This approach strengthens the robustness of the proposed fraud detection system by justifying the selection of methodologies and hyperparameters. It uses advanced machine learning to improve detection accuracy and reduce financial fraud risks. Subsequent sections discuss every module of the framework in detail.

**Algorithm 1. Proposed FFD algorithm pesudocode.**

1: **Input:** Raw transaction data from European cardholders (ECH Dataset 2023) and IEEE-CIS dataset

2: **Output:** Model for detecting fraudulent transactions

3: **Step 1: Data Preprocessing**

4: Load datasets: ECH and IEEE-CIS

5: **for** each dataset **do**

6:   Fill missing values

7:   Detect and eliminate outliers

8:   Normalize features

9:   Standardize features

10:   Encode categorical labels

11: **end for**

12: **Step 2: Data Balancing using SMOTE**

13: Identify minority class samples

14: **for** each minority class sample **do**

15:   Find *k* nearest neighbors

16:   **for** each neighbor **do**

17:    Generate synthetic sample along the line segment connecting to the neighbour

18:   **end for**

19: **end for**

20: Add synthetic samples to the dataset to achieve a balanced class distribution

21: Step 3: Feature Engineering

22: Feature Selection:

23: Compute Pearson correlation coefficient

24: Select features with a strong correlation

25: Feature Creation:

26: **for** each domain-specific heuristic **do**

27:   Generate new features

28: **end for**

29: Step 4: Build RMGACNet Model

30: BiLSTM Layer: Extract features from sequential data using BiLSTM

31: GraphSAGE Layer with L2 Regularization: Learn node embeddings and apply L2 regularization

32: Capsule Layer with L2 Regularization: Apply Capsule Layer and L2 regularization

33: Attention Mechanism with L2 Regularization: Apply Attention Mechanism and L2 regularization

34: Graph Attention Networks (GATs): Apply GATs to extract complex graph representations

35: Step 5: Model Training and Evaluation

36: Train RMGACNet model on the balanced and preprocessed dataset

37: Evaluate the model performance using metrics

### Dataset description

#### European Card Holder (ECH) dataset 2023

Credit card transactions from European cardholders in 2023 were utilized for this study [24]. More than 568,630 entries with V1–V28 transaction characteristics are included. These components describe time, location, and transaction type, among others. The dataset also has a binary label indicating if the transaction is fraudulent and its value. By anonymizing the dataset, attackers cannot identify cardholders. It is a valuable resource for creating algorithms and models to identify fraudulent transactions. Academics can analyze credit card transactions, merchant types, and more using this information. European cardholders’ 2023 credit card transactions form this dataset as shown in Table 2. Sensitive material was removed for ethical and privacy reasons.

**Table 2:** ECH Dataset 2023 (Header view).

id	V1	V2	V3	V4	...	...	V26	V27	V28	Amount	Class
0	–0.261	–0.470	2.496	–0.084	...	...	–0.435	–0.081	–0.151	17982.100	0
1	0.985	–0.356	0.558	–0.430	...	...	0.297	–0.248	–0.065	6531.370	0
2	–0.260	–0.949	1.729	–0.458	...	...	–0.313	–0.300	–0.245	2513.540	1
3	–0.152	–0.509	1.747	–1.090	...	...	–0.516	–0.165	0.048	5384.440	0
4	–0.207	–0.165	1.527	–0.448	...	...	1.071	0.024	0.419	14278.970	1
5	0.025	–0.141	1.191	–0.708	...	...	0.253	0.067	0.096	6901.490	0
6	1.016	–0.397	0.498	–0.144	...	...	–0.604	–0.198	–0.088	18954.450	1

#### IEEE CIS dataset

Table 3 presents a well-organized sample of financial transactional data from the IEEE-CIS fraud detection dataset [25]. The collection contains unique TransactionIDs for each transaction. The dataset has 590540 records and 394 characteristics. Transaction details include the fraud flag (isFraud), timestamp (TransactionDT), amount (TransactionAmt), product code (ProductCD), and card-related details like card type (card6), address information (addr1 and addr2), and distance measures (dist1 and dist2).

Also in the collection are numerical features designated C1 to C14, D1 to D15, M1 to M9, and V1 to V339. These parameters may include transaction number, timing discrepancies, matching status, and aggregated transaction data.

**Table 3:** IEEE-CIS dataset sample.

TransactionID	isFraud	TransactionDT	TransactionAmt	ProductCD	card1	...	addr1	C1	...	V339
2987000	0	86400	68.5	W	13926	...	315	1	...	0
2987001	0	86401	29	W	2755	...	325	1	...	0
2987002	0	86469	59	W	4663	...	330	1	...	0
2987003	1	86499	50	W	18132	...	476	2	...	0
2987004	1	86506	50	H	4497	...	420	1	...	0
2987005	1	86510	49	W	5937	...	272	1	...	0
2987006	0	86522	159	W	12308	...	126	1	...	0
2987007	0	86529	422.5	W	12695	...	325	1	...	0
2987008	1	86535	15	H	2803	...	337	1	...	0
2987009	1	86536	117	W	17399	...	204	2	...	0
2987010	1	86549	75.887	C	16496	...	325	1	...	0
2987011	1	86555	16.495	C	4461	...	0	30	...	0

### Data preprocessing

The suggested system includes critical preprocessing operations to enhance input data quality and consistency [26]. In circumstances of missing data, employ missing value imputation. Mean imputation is often used for numerical characteristics *x*_*i*_, replacing missing values *NaN* with the average (μ) of non-missing values. This equation describes this method:

$$x_{i}=\frac{\sum_{j=1}^{N}x_{j}}{N}$$ (1)

In categorical features, the mode, or most prevalent value, substitutes the absent value. Outlier detection and elimination are used to exclude observations with extreme values. The formula for determining Z-scores (V) for quantitative attributes is as follows [26,27]:

$$V=\frac{c_{u}-\bar{c}}{\sigma }$$ (2)

( c_i ), the mean is ( barc ), and the standard deviation is ( sigma ). Observations having z-scores beyond a specific threshold—such as v>3 or v<−3—are noted as outliers and deleted from the dataset. Numerical data are normalized and standardized after outlier reduction to guarantee consistency. Min-max scaling standardizes features to 0–1 mean and standard deviation using 0–1 normalizing range. One may find the normalizing and standardizing equations in [26].

$$x_{\text{normalized}}=\frac{x_{i}-\text{min}(x)}{\text{max}(x)-\text{min}(x)}$$ (3)

$$x_{\text{standardized}}=\frac{x_{i}-\mu }{\sigma }$$ (4)

Label encoding is also used to convert numerical variables from category ones. A unique integer value between 0 and *K* − 1 is assigned to each category, with *K* indicating the total number of different categories. The input data is improved, standardized, and encoded appropriately before analysis and model training.

### Data balancing using SMOTE algorithm

Using the Synthetic Minority Over-sampling Technique (SMOTE), class imbalances may be corrected, and the model trained on a balanced representation of both classes [28]. SMOTE uses existing features to create synthetic samples for underrepresented groups to attain class parity. The algorithm’s steps are:

- First, we need to find the datasets that include samples from the minority class.
- Neighborhoods: In the feature space, the *k* closest neighborhoods are found for every minority class sample.
- The line segments that link the samples of minority classes to their nearest neighbours are randomly selected to create synthetic samples.
- Dataset with Equal Representation of Both Classes: A balanced dataset is created by supplementing the original dataset with these synthetic examples.

To create synthetic samples, the following equation is used [28]:

Synthetic Sample=Sample+random×(Nearest Neighbor−Sample) (5)

**Fig 2:**
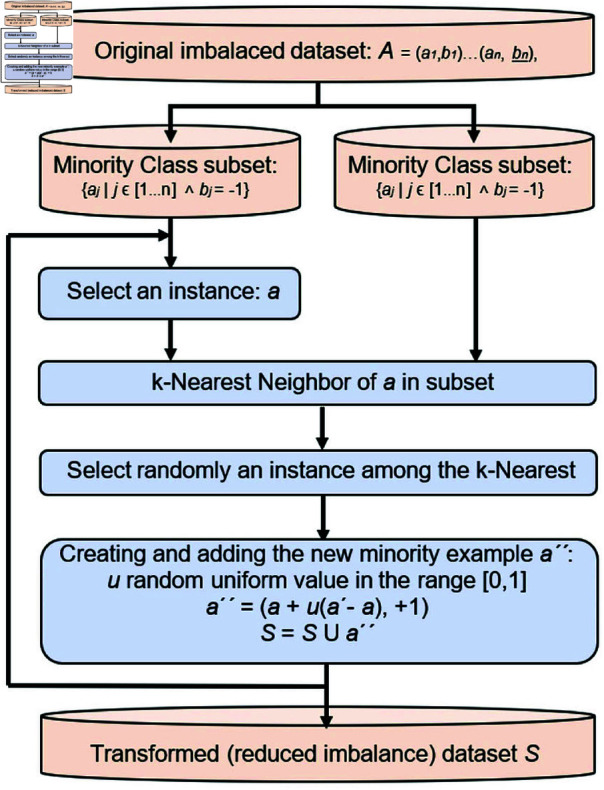
Flowchart data balancing algorithm (SMOTE).

When a formulated number between zero and one is used, the SMOTE method is used to level the dataset, improving the model’s predictive power. This ensures that the dataset is representative and objective, which is crucial for training the model. The SMOTE method may be shown in Fig 2.

### Feature engineering

Feature engineering enhances machine learning models’ prediction ability, notably in FFD. This section covers two crucial aspects of feature engineering: selection and extraction/creation [29,30].

Feature selection identifies and eliminates the most predictive components of the model. Feature selection is often conducted using feature correlation analysis, which examines the direction and strength of the linear connection between characteristics and the target variable. The conventional method uses the Pearson correlation coefficient (ρ).

$$\rho_{X,Y}=\frac{\text{cov}(X,Y)}{\sigma_{X}\sigma_{Y}}$$ (6)

The covariance of feature *X* and target variable *Y* is indicated as cov(X,Y), while the standard deviations of *X* and *Y* are represented by $\sigma_{X}$ and $\sigma_{Y}$, respectively. To train the model, features are retained with high correlations to the target variable (absolute correlation coefficients close to 1 or –1) and exclude features with weak correlations.

### Proposed Regularized Memory Graph Attention CapsuleNet (RMGACNet) model

**Fig 3:**
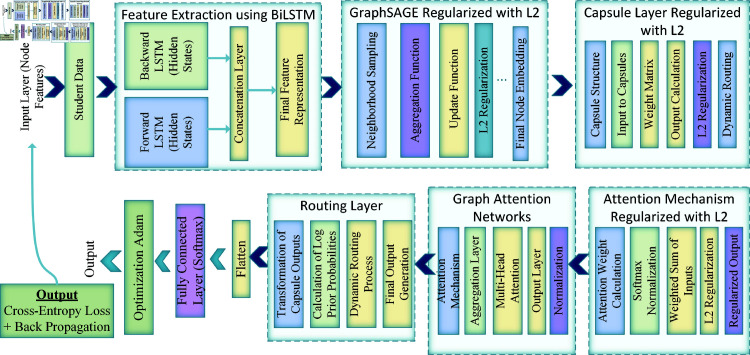
RMGACNet model architecture.

The RMGACNet model uses deep learning, graph representation learning, and capsule networks to identify financial fraud. The RMGACNet design uses memory mechanisms, graph attention mechanisms, and capsule networks to record complicated financial transaction data linkages and patterns. See the architecture in Fig 3.

#### Feature extraction using BiLSTM

The model employs BiLSTM networks to extract features from sequential financial transaction data, enabling it to recognize patterns and correlations across time [31]. BiLSTM networks manage forward and backward transaction sequences to let the model learn from the past and future. The following equations regulate all BiLSTM networks:

Forward LSTM:

$$\alpha_{t}^{f}=\sigma (\Theta_{xi}^{f}x_{t}+\Theta_{hi}^{f}h_{t-1}^{f}+b_{i}^{f})\;\beta_{t}^{f}=\sigma (\Theta_{xf}^{f}x_{t}+\Theta_{hf}^{f}h_{t-1}^{f}+b_{f}^{f})\;\gamma_{t}^{f}=\tanh(\Theta_{xg}^{f}x_{t}+\Theta_{hg}^{f}h_{t-1}^{f}+b_{g}^{f})\;\delta_{t}^{f}=\sigma (\Theta_{xo}^{f}x_{t}+\Theta_{ho}^{f}h_{t-1}^{f}+b_{o}^{f})\;\epsilon_{t}^{f}=\beta_{t}^{f}⊙\epsilon_{t-1}^{f}+\alpha_{t}^{f}⊙\gamma_{t}^{f}\;\zeta_{t}^{f}=\delta_{t}^{f}⊙\tanh(\epsilon_{t}^{f})$$ (7)

Backward LSTM:

$$\alpha_{t}^{b}=\sigma (\Theta_{xi}^{b}x_{t}+\Theta_{hi}^{b}h_{t+1}^{b}+b_{i}^{b})\;\beta_{t}^{b}=\sigma (\Theta_{xf}^{b}x_{t}+\Theta_{hf}^{b}h_{t+1}^{b}+b_{f}^{b})\;\gamma_{t}^{b}=\tanh(\Theta_{xg}^{b}x_{t}+\Theta_{hg}^{b}h_{t+1}^{b}+b_{g}^{b})\;\delta_{t}^{b}=\sigma (\Theta_{xo}^{b}x_{t}+\Theta_{ho}^{b}h_{t+1}^{b}+b_{o}^{b})\;\epsilon_{t}^{b}=\beta_{t}^{b}⊙\epsilon_{t+1}^{b}+\alpha_{t}^{b}⊙\gamma_{t}^{b}\;\zeta_{t}^{b}=\delta_{t}^{b}⊙\tanh(\epsilon_{t}^{b})$$ (8)

*x*_*t*_, the hidden states of the ahead and backward LSTMs are denoted by $\zeta_{t}^{f}$, $\zeta_{t}^{b}$ respectively, and the cell states of the ahead and backwards LSTMs are denoted by $\epsilon_{t}^{f}$, $\epsilon_{t}^{b}$ respectively. Notably, the symbols $\alpha_{t}^{f}$, $\beta_{t}^{f}$, $\gamma_{t}^{b}$, and $\delta_{t}^{f}$, respectively denote the input gate, forget gate, cell gate, and output gate for the forward and reverse LSTMs. Component-wise multiplication is symbolized by the ⊙ symbol.

Due to its adeptness in identifying relationships and sequential patterns in financial transaction data, BiLSTM networks provide the RMGACNet model with valuable information for further processing.

#### GraphSAGE regularized with L2

Node embeddings in graph-structured data are learnt using GraphSAGE Layer [32]. GraphSAGE builds node embeddings using samples from surrounding graph nodes. The GraphSAGE model is trained using L2 regularization to improve generalization and reduce overfitting. To use GraphSAGE with L2 regularization, the following equations must be met [32]:

$${𝐡}_{v}^{k}=\text{AGGREGATE}\left(\{{𝐡}_{u}^{k-1},\forall u\in 𝒩(v)\}\right)\;{𝐡}_{v}^{k}=\text{ReLU}\left({𝐖}^{k}\cdot \text{CONCAT}({𝐡}_{v}^{k-1},{𝐡}_{v}^{k})\right)\;{𝐡}_{v}^{k}=\text{L2\_REGULARIZATION}({𝐡}_{v}^{k})$$ (9)

In this equation, ${𝐡}_{v}^{k}$ denotes the embedding of node v at layer *k*, 𝒩(v) signifies the neighbouring nodes, ${𝐖}^{k}$ indicates the weight matrix, and AGGREGATE refers to a neighbourhood aggregation function. The main methods include ReLU activation after aggregation and implementing L2 regularisation to mitigate excessive weights and avert overfitting.

GraphSAGE with L2 regularization improves the RMGACNet model’s ability to capture structural information from financial transaction networks and reduces overfitting.

#### Capsule layer regularized with L2

The RMGACNet Capsule Layer holds hierarchical links and input data feature settings. Each capsule, which stands for a different object or attribute, is defined by instantiation parameters. L2 regularising of instantiation parameters lowers overfitting and improves Capsule Layer generalization. Operating with L2 regularisation, the Capsule Layer has to meet the following equations [33]:

$$z_{mn}=R_{mn}\cdot p_{m}\;t_{n}=\sum_{m}\beta_{mn}\cdot z_{mn}\;t_{n}=\text{ACTIVATE}(t_{n})\;t_{n}=\text{L2\_REGULARIZATION}(t_{n})$$ (10)

$z_{mn}$ denotes the input of capsule *n* from capsule *m*, $p_{m}$ signifies the output of capsule *m* from the preceding layer, $R_{mn}$ indicates the weight matrix linking capsule *m* to capsule *n*, $\beta_{mn}$ represents the coupling coefficients, and ACTIVATE(·) pertains to the activation function.

Imposing L2 regularisation on the instantiation parameters of the Capsule Layer reduces overfitting by penalizing high parameter values, promoting the acquisition of more generalizable features within the input data.

#### Attention mechanism regularized with L2

The RMGACNet model relies on the Attention Mechanism layer to concentrate on important data and discard irrelevant data. Input data components are given attention weights to help the model concentrate on key traits. L2 regularization of attention weights improves the Attention Mechanism and reduces overfitting. The following equations govern the Attention Mechanism with L2 regularization, as stated in [33,34]:

$${𝐳}_{j}=\text{ATTENTION\_WEIGHTS}({𝐩}_{j},{𝐐}_{b})\;{𝐛}_{j}=\text{SOFTMAX}({𝐳}_{j})\;𝐯=\sum_{j}{𝐛}_{j}\cdot {𝐩}_{j}\;𝐯=\text{L2\_REGULARIZATION}(𝐯)$$ (11)

${𝐩}_{j}$ represents the input attributes for the j-th occurrence. Attention scores are calculated using the attention weight matrix ${𝐐}_{b}$. Attention ratings for each occurrence are indicated by ${𝐳}_{j}$. Attention weights are indicated by ${𝐛}_{j}$ after softmax normalization of attention scores. To reduce overfitting, L2 regularization is used for the final attended output features (𝐯).

#### Graph attention networks

Graph Attention Networks (GATs) are robust neural networks that handle graphs. They can record complex graph connections and relationships. GATs are utilized in RMGACNet to extract complicated input data representations while preserving graph structure. Since corporate transactions can be graphed, this helps identify financial fraud. The equations governing Graph Attention Networks are as follows [35]:

$${𝐡}_{i}^{\left(l\right)}=\sigma \left(\sum_{j\in 𝒩(i)}\beta_{ij}^{\left(l\right)}\cdot {𝐕}^{\left(l\right)}\cdot {𝐡}_{j}^{\left(l-1\right)}\right)\;\beta_{ij}^{\left(l\right)}=\frac{\text{exp}(\text{LeakyReLU}({𝐳}^{(l)T}[{𝐕}^{\left(l\right)}\cdot {𝐡}_{i}^{\left(l-1\right)}||{𝐕}^{\left(l\right)}\cdot {𝐡}_{j}^{\left(l-1\right)}]))}{\sum_{k\in 𝒩(i)}\text{exp}(\text{LeakyReLU}({𝐳}^{(l)T}[{𝐕}^{\left(l\right)}\cdot {𝐡}_{i}^{\left(l-1\right)}||{𝐕}^{\left(l\right)}\cdot {𝐡}_{k}^{\left(l-1\right)}]))}\;{𝐡}_{i}^{\left(l\right)}=\text{L2\_REGULARIZATION}({𝐡}_{i}^{\left(l\right)})$$ (12)

The concealed representation of node *i* at layer *l* is represented as ${𝐡}_{i}^{\left(l\right)}$. The vicinity of node *i* is denoted as 𝒩(i). The attention coefficient between nodes *i* and *j* at layer *l* is represented as $\beta_{ij}^{\left(l\right)}$. The weight matrix at layer *l* is denoted as ${𝐕}^{\left(l\right)}$. The variables of the attention mechanism are represented as ${𝐳}^{\left(l\right)}$. The activation function is denoted as σ(·). The RMGACNet model uses Graph Attention Networks to effectively capture the relational information in the input data, hence improving performance in FFD tasks.

#### Routing layer

The Routing Layer of the RMGACNet model enhances capsule layer predictions. Dynamic routing lets capsules expressing the same object or feature at many abstraction levels interact and agree. This layer increases the model’s discriminative ability to generate robust, instructive representations. Dynamic routing technology of Capsule Networks is used in the RMGACNet concept. As long as they agree, the technique will update coupling coefficients between lower- and higher-level capsule predictions. For a general routing process, following equation is used [36].

$${𝐩}_{kl}={𝐌}_{kl}\cdot {𝐮}_{k}\;{𝐪}_{kl}=\text{softmax}({𝐩}_{kl})\;{𝐫}_{l}=\text{softmax}({𝐪}_{kl}\cdot {𝐮}_{k})$$ (13)

In this instance, ${𝐮}_{k}$ denotes the output of capsule *k*, ${𝐌}_{kl}$ signifies the transformation matrix between capsule *k* and capsule *l*, ${𝐩}_{kl}$ represents the logarithmic prior probabilities, ${𝐪}_{kl}$ pertains to the coupling coefficients, and ${𝐫}_{l}$ indicates the output of capsule *l*.

Using recurring agreement adjustments, capsules may approach a consensus on their predictions and thereby enhance the stability and accuracy of their representations using the dynamic routing technique. Thus, because it can understand the hierarchical linkages and relationships in the input data, the RMGACNet model is more suited to detect financial fraud.

#### Aggregation layer

The RMGACNet model’s aggregate layer finishes the analysis after compiling data from abstraction and capsule layers. High-level feature representation, including the prominent features of the input, results from the merging of capsule layer outputs with routing process contextual information. Aggregation is the gathering and combining of data from various sources. This layer needs forecasts from many network segments to enable informed decision-making for activities farther down the pipeline. Aggregation may be shown mathematically as follows:

$${𝐲}_{k}=\text{combine}({𝐠}_{k})$$ (14)

${𝐠}_{k}$, represents the output of capsule *k*; ${𝐲}_{k}$ combines the representations produced by aggregating the outputs of the capsules. The aggregation function used by the RMGACNet model may vary based on the task and the characteristics of the incoming data. Standard aggregating functions include max pooling, mean pooling, and attention-based aggregation. The Aggregation Layer extracts valuable data from the capsule outputs via these actions, offering a succinct but informative representation for further processing.

#### Output layer

The output layer of the RMGACNet model makes predictions using aggregated representations from the aggregagement layer. It forecasts, on aggregated features, the target variable or variables using classification or regression techniques. FFD calls on the output layer to use learnt representations to determine if a transaction or event is fraudulent. The output forecast mathematically is:

$$\hat{j^{\prime }}=\text{Output}(g^{\prime })$$ (15)

Where the projected output is indicated $\hat{g^{\prime }}$ and the aggregated representation produced from the Aggregation Layer is marked $j^{\prime }$. $\dot{\text{T}}$he Output Layer determines whether a transaction is fraudulent using FFD-specific categorization methods. Further analysis and interpretation of the anticipated results using performance criteria help identify suspected fraud occurrences.

### Hyperparameter tuning process

The Spotted Hyena Optimiser (SHO) efficiently navigates complicated search regions. RMGACNet hyperparameters were changed. Model performance depends on learning rate, layer count, batch size, and dropout rates. Based on spotted hyena hunting techniques, the SHO methodology explores the hyperparameter space and converges on appropriate configurations. Sequential hyperparameter optimization assessed model performance after tweaking several hyperparameter values. The model is tuned for accuracy and F1 score, determining its positive and negative classification capacity. Using k-fold cross-validation ensures hyperparameter selection values are generalized across data subsets, reducing overfitting. For ideal hyperparameter settings, Table 4 after many SHO repeats. This table shows the chosen hyperparameters and their performance measurements, confirming our tuning method. The SHo changes RMGACNet’s model architecture, making it more scalable and applicable to real-time financial applications.

**Table 4:** Optimal hyper parameters for RMGACNet.

Hyperparameter	Optimal Value
Activation Function	ReLU
Learning Rate	0.001
Number of Layers	4
Epochs	100
Dropout Rate	0.3
Optimizer	Adam
Batch Size	32

## Simulation and results

The following requirements are applied to a system that undergoes a rigorous simulation process: 32 GB of RAM, a GeForce RTX 2080Ti GPU, and a 2.4 GHz Dell Core i7 12th generation quad-core CPU. Several Python libraries, including NumPy, PyTorch, and Pandas, are used in the simulations. The GPU is used for both the model’s training and evaluation processes. The Adam Optimiser modifies the model’s parameters during the training process. The ECH and IEEE-CIS datasets include an initial learning rate of 0.001. The batch size is set at 512. The model is trained on both datasets until convergence, using a maximum of 120 epochs.

The dataset was thoroughly analysed for missing values, anomalies, data normalization, and standardization. This preliminary study obtained a pristine dataset for analysis. Missing data is addressed to avert bias and mistakes in the study. Outliers were identified and addressed to prevent prejudice. Data normalization and standardization standardized all characteristics to improve the performance and convergence of machine learning models. This comprehensive preprocessing was necessary to ensure the accuracy of analysis and model development. The data is analysed by correlation.

**Fig 4:**
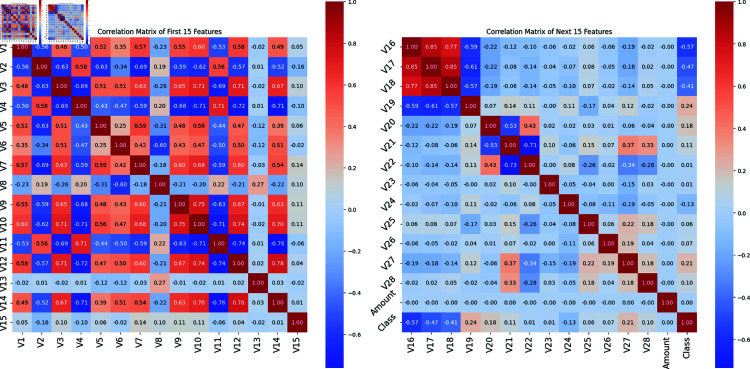
Data correlation of all features (ECH dataset).

Fig 4, left figure, shows the correlation matrix for the first 15 criteria, including the ‘Class’ column indicating fraudulent transactions. V12, V10, and V14 are essential fraud detectors due to their substantial positive correlations with ‘Class’ (0.58, 0.60, and 0.49). V4 and V12 have a strong negative correlation of –0.72, whereas V3 and V10 have a 0.71 positive connection. V2 and V7 also correlate –0.69. These findings identify essential collection features and probable duplication. The association matrix for the other 15 attributes is illustrated on the right side of the figure. V16, V17, and V18 show the most negative correlations (–0.57, –0.47, and –0.41) with ‘Class’, suggesting a solid inverse link with fraud. Meaningful interactions include a strong positive correlation (0.85) between V16 and V17 and (0.77) between V16 and V18. The correlation value of –0.19 suggests a poor link between V24 and V27. Fraud detection models’ features and multicollinearity management depend on these interactions. It also highlights essential traits highly associated with ‘Class’ and interactions between features. This information helps create fraud detection tools.

**Fig 5:**
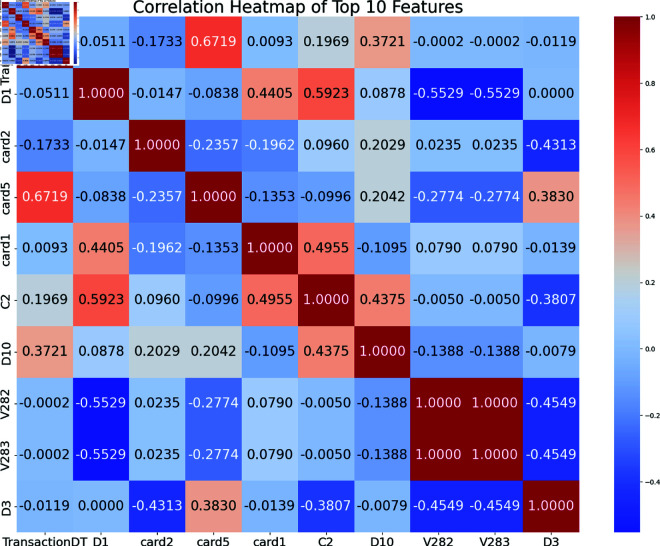
Data correlation of top 10 features (IEEECIS dataset).

A correlation heatmap of the top ten indicators used to identify financial fraud from the IEEE-CIS dataset is shown in Fig 5. Correlation coefficients between pairs of attributes are shown in this heatmap from –1 (totally negative correlation) to 1 (totally positive correlation). Card5 is strongly positively correlated with TransactionDT (0.6719), D10 is moderately positively correlated with it (0.3721), and card2 is somewhat negatively correlated with it (–0.1733). Correlations between the D1 trait and C2(0.5923) and card1(0.4455) are positive, whereas V282 and V283 are negatively associated (–0.5529). The C2 characteristic is positively associated with D10 (0.4375) but negatively associated with D3 (–0.3807). The card5 feature correlates positively with D10 (0.2042) and card1 (0.3830) and negatively with V282 and V283 (–0.2774). V282 and V283 exhibit a perfect correlation (1.0000) and strong negative correlations with D3 (–0.4549 each).

**Fig 6:**
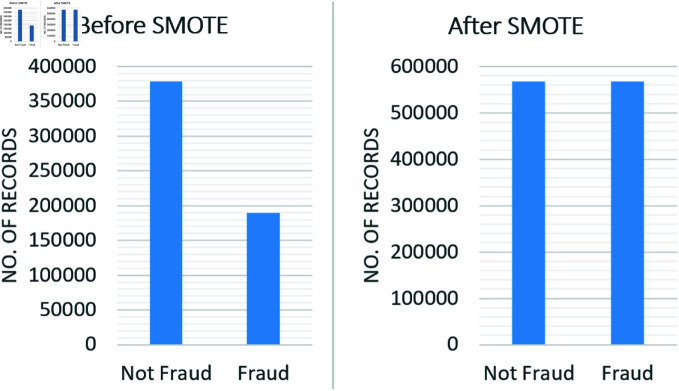
Data before and after applying SMOTE algorithm.

Fig 6 for dataset records before and after the SMOTE technique application. ‘Not fraud’ cases initially outnumber ‘fraud’ ones considerably. The dataset becomes balanced when SMOTE is applied, with equal records in each class. A balance between training machine learning models on ‘fraud’ and ‘non-fraud’ situations improves their accuracy and dependability in recognizing fraudulent transactions.

Our framework uses BiLSTM for feature selection/extraction. Features V1, V3, V4, V7, V9, V10, V11, V12, and V14 show fraud patterns. These features score high in the BiLSTM network, indicating their importance in categorization. The BiLSTM model correctly detects substantial negative correlations between the ‘Class’ variable and characteristics V16, V17, V18, and V19, demonstrating their importance in identifying fraud. Because of their contribution, the BiLSTM network can discover nuanced patterns and behaviours, improving the model’s prediction capacity. Thus, the fraud detection model has characteristics V21–V27. The BiLSTM model performs well in assessing the ‘Amount’ feature, which assesses transaction value and fraud risk. The model’s relevance in research is highlighted. Table 5 displays the chosen features and their scores.

**Table 5:** Selected features and their BiLSTM scores on ECH dataset (Sfeat: selected feature).

Sfeat	Score	Sfeat	Score	Sfeat	Score
V1	0.82	V12	0.91	V22	0.70
V3	0.88	V14	0.86	V23	0.68
V4	0.85	V16	0.80	V24	0.74
V7	0.90	V17	0.78	V25	0.73
V9	0.87	V18	0.75	V27	0.76
V10	0.89	V19	0.77	Amount	0.81
V11	0.84	V21	0.72		

**Fig 7:**
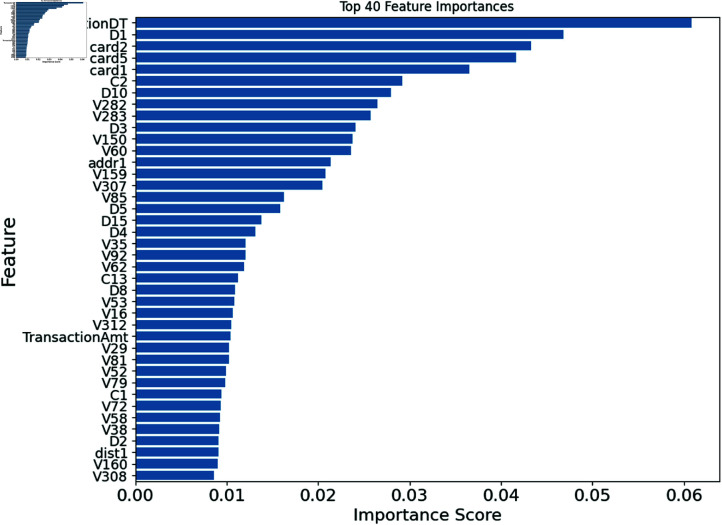
Top 40 features selected by BiLSTM from IEEECIS dataset.

Fig 7 displays the top 40 features selected by a BiLSTM model from the IEEECIS dataset. These 40 features predicted the target variable the most out of 339 in the dataset. These attributes’ significance scores were determined during model training, with higher scores suggesting a more significant effect on target variable prediction. The importance ratings for qualities are also shown. The length of each bar shows the BiLSTM model’s relevance score for that feature. Features with longer bars predict the target variable better than those with shorter bars.

**Table 6:** Proposed framework performance values on the ECH dataset.

Metric	RMGACNet	ResNeXt-GRU [6]	LSTM [22]	GhostNet [6]	DCNN [15]	SVM [10]	GANs [17]
Precision	0.9772	0.8683	0.8571	0.8782	0.8205	0.8302	0.9235
Log Loss	0.2259	0.3312	0.3423	0.3198	0.4025	0.3898	0.3012
Accuracy	0.9768	0.8623	0.8534	0.8671	0.8064	0.8181	0.9323
G-mean	0.9834	0.9113	0.8956	0.9157	0.8632	0.8734	0.9321
Kappa statistic	0.9769	0.8621	0.8505	0.8715	0.8153	0.8248	0.9172
Recall	0.9769	0.8656	0.8525	0.8715	0.8124	0.8221	0.9479
Specificity	0.9768	0.9156	0.9032	0.9224	0.8695	0.8811	0.8976
F1 Score	0.9770	0.8665	0.8548	0.8745	0.8167	0.8267	0.9362
MCC	0.9770	0.8612	0.8503	0.8721	0.8145	0.8242	0.8467
Average Precision	0.9771	0.8715	0.8598	0.8802	0.8326	0.8421	0.9254
Balanced Accuracy	0.9770	0.8925	0.8773	0.8950	0.8444	0.8508	0.9157
AUC-PR	0.9860	0.8740	0.8610	0.8830	0.8300	0.8400	0.9300

**Table 7:** Proposed Framework Performance values on the IEEE-CIS dataset.

Metric	RMGACNet	ResNeXt-GRU [6]	LSTM [22]	GhostNet [6]	DCNN [15]	SVM [10]	GANs [17]
Precision	0.9882	0.8791	0.8653	0.8912	0.8347	0.8462	0.9331
Log Loss	0.2412	0.3487	0.3551	0.3318	0.4125	0.4052	0.3113
Accuracy	0.9876	0.8704	0.8605	0.8792	0.8223	0.8287	0.9421
G-mean	0.9943	0.9206	0.9109	0.9267	0.8793	0.8831	0.9402
Kappa statistic	0.9872	0.8709	0.8615	0.8726	0.8321	0.8319	0.9317
Recall	0.9887	0.8753	0.8668	0.8781	0.8269	0.8312	0.9568
Specificity	0.9862	0.9265	0.9178	0.9345	0.8887	0.8964	0.9142
F1 Score	0.9879	0.8798	0.8672	0.8893	0.8326	0.8401	0.9463
MCC	0.9876	0.8736	0.8642	0.8781	0.8296	0.8385	0.8602
Average Precision	0.9881	0.8825	0.8703	0.8918	0.8413	0.8502	0.9364
Balanced Accuracy	0.9865	0.9236	0.9124	0.9218	0.8837	0.8914	0.9264
AUC-PR	0.9900	0.8850	0.8730	0.8980	0.8440	0.8505	0.9470

The tables describe the performance of multiple models on two financial fraud identification datasets: the European cardholder (ECH) transactions dataset and the IEEE-CIS dataset. The results also demonstrate that the suggested RMGACNet model is more effective than the most advanced, state-of-the-art FFD approaches, which bodes well for its practical use. The proposed RMGACNet model’s performance metrics are compared to current state-of-the-art models on the ECH dataset in Table 6. RMGACNet has good results in Precision(0.9772), Accuracy(0.9768), and F1 Score (0.9770). With a Log Loss of just 0.2255, RMGACNet more reliably estimates probabilities than any other network. The capacity of RMGACNet to maintain a balanced ratio of true positives to true negatives is shown by its highest Kappa statistic (0.9769) and G-mean (0.9834) values. Table 7 displays the performance metrics of several benchmark models on the IEEE-CIS dataset, including the proposed RMGACNet model. RMGACNet performs well with Precision, Accuracy, and F1 Score values of 0.98821, 0.98821, and 0.98821, respectively. Its highest G-mean (0.99521) and Kappa statistic (0.98821) among the evaluated models demonstrate its robust performance.

**Table 8:** Statistical analysis tests on IEEE-CIS and ECH dataset.

Techniques	Wilcoxon	Spearman’s	Chi-Squared	Paired Student	ANOVA	Studenta’s
RMGACNet (F-stat)	7.632	0.822	173	0.04	49.305	0.00
RMGACNet (P-val)	0.022	0.005	–0.010	0.001	0.000	0.060
ResNeXt-GRU (F-stat)	6.710	0.664	214	–4.775	56.977	–1.914
ResNeXt-GRU (P-val)	0.031	0.019	–0.010	0.000	0.001	0.031
LSTM (F-stat)	7.209	0.735	191	–4.133	51.753	–1.642
LSTM (P-val)	0.019	0.012	–0.010	0.000	0.000	0.044
GhostNet (F-stat)	7.913	0.800	179	–3.905	47.914	–1.355
GhostNet (P-val)	0.027	0.008	–0.010	0.000	0.000	0.055
DCNN (F-stat)	5.973	0.522	285	–5.325	62.173	–2.488
DCNN (P-val)	0.039	0.033	–0.010	–0.010	0.000	0.026
SVM (F-stat)	6.375	0.602	239	–4.991	58.410	–2.099
SVM (P-val)	0.032	0.026	–0.010	0.000	0.001	0.039
GANs (F-stat)	5.310	0.448	325	–6.249	68.915	–2.997
GANs (P-val)	0.045	0.044	–0.010	–0.010	0.000	0.017

Table 8 compares the RMGACNet model to top FFD methods using statistical analysis on IEEE-CIS and ECH datasets. Wilcoxon, Spearman’s, Chi-Squared, Paired Student, ANOVA, and Student Tests produce F-statistics and P-values for each model. RMGACNet, the proposed model, has Wilcoxon (F-stat: 7.632, P-val: 0.022), Spearman’s (F-stat: 0.822, P-val: 0.005), Chi-Squared (F-stat: 173, P-val: –0.010), Paired Student (F-stat: 0.04, P-val: 0.001), ANOVA (F-stat: 49.305, P-val: 0.000), and Student values. RMGACNet’s excellent F-statistics, significant P-values, and predictive abilities show it performs well in most tests. ResNeXt-GRU, LSTM, GhostNet, DCNN, SVM, and GANs perform differently. On IEEE-CIS and ECH datasets, RMGACNet regularly beats these models, making it the most reliable and effective financial misbehaviour detection approach.

**Fig 8:**
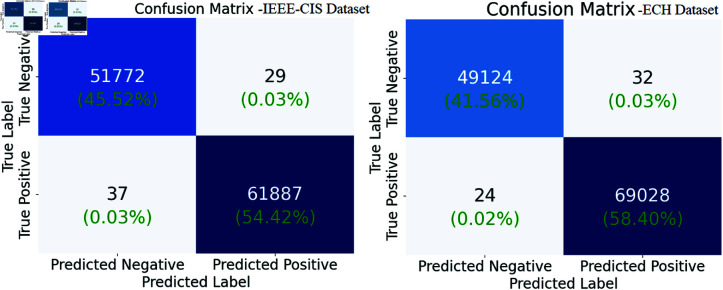
Confusion matrices of RMGACNet on ECH and IEEE-CIS datasets.

Fig 8 show the confusion matrices of the RMGACNet model on the IEEE-CIS and ECH datasets. This shows the model can recognize fraudulent and non-fraudulent transactions. The model accurately identifies a substantial number of true negatives (49,124) and true positives (69,028) with minimal false positives (32) and false negatives (24), indicating good detection capability (Left, IEEE-CIS). Similarly, on the right side of the figure (ECH), RMGACNet achieves high true negative (51,772) and true positive (61,887) counts with low false positives (29) and negatives (37). The model accurately and reliably detects financial fraud in both datasets with low misclassification rates.

**Fig 9:**
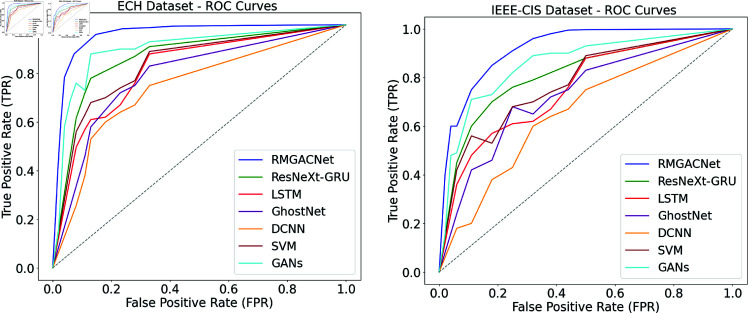
ROC curves comparison of proposed and existing methods.

A comparison is made between the RMGACNet model and current approaches utilizing the ROC curve to assess its FFD on the ECH dataset in Fig 9. As a function of threshold value, the ROC curve plots the ratio of true positives (Sensitivity) to false positives (1-Specificity). A higher AUC indicates a more accurate separation of positive and negative categories. RMGACNet’s ROC curve is better than others, showing it can consistently identify fraudulent transactions. A practical FFD tool, RMGACNet makes more accurate predictions with fewer false positives and negatives.

**Fig 10:**
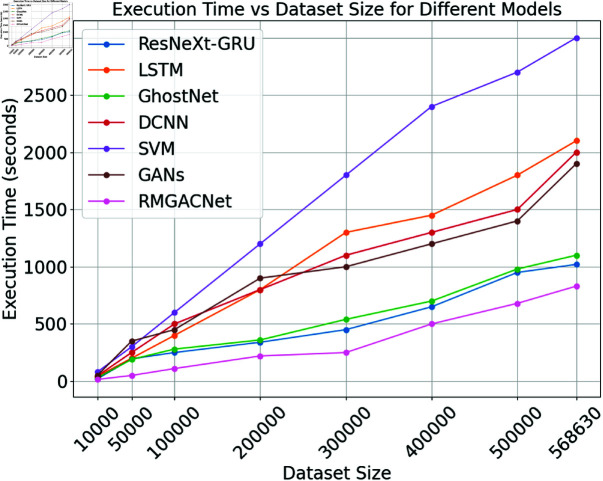
Execution time of classifiers.

Fig 10 compares the performance duration of several models (ResNeXt-GRU, LSTM, GhostNet, DCNN, SVM, GANs, and RMGACNet) on various dataset sizes. On the x-axis, there are 10,000 to 568,630 data points. The y-axis shows seconds of execution. As dataset quantities change and execution times rise, model performance is monitored. RMGACNet always executes the quickest, from 15 seconds for the smallest dataset to 830 seconds for the largest. Compared to other ways, it is effective and increasing. The upgraded RMGACNet integrates feature extraction and classification to reduce computational complexity and speed processing. ResNeXt-GRU and GhostNet execute quickly, but RMGACNet does better. SVM and GANs take the longest to perform on larger datasets, whereas LSTM and DCNN are faster. The SVM takes 3000 seconds on the largest dataset, showing its inefficiency with large data sets.

**Fig 11:**
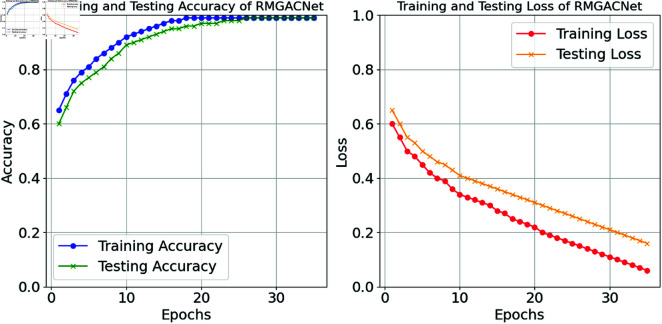
RMGACNet model training accuracy loss analysis.

Several regularisation techniques are used to evaluate the risk of overfitting in the RMGACNet model to improve generalization abilities. Notwithstanding the model’s intricate construction, our findings demonstrate a robust correlation between training and testing performance, as seen in Fig 11. The close alignment of training and testing accuracy and the steady decline in training and testing loss indicate successful mitigation of overfitting risk. Different strategies like dropout layers and L2 regularisation are used to enhance the stability of the learning process and mitigate excessive model complexity. This research affirms our dedication to maintaining RMGACNet’s robustness in real-world contexts, validating its dependability for practical applications in financial fraud detection.

**Fig 12:**
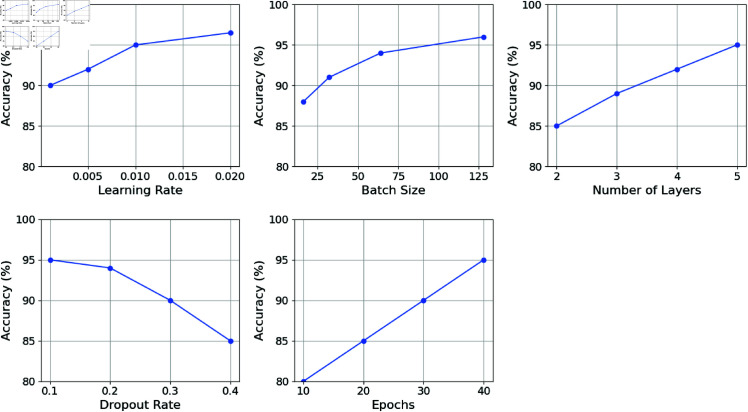
RMGACNet parameter sensitivty analysis.

Fig 12 shows the RMGACNet model’s hyperparameter sensitivity analysis and its effect on accuracy. The graph has five hyperparameter subplots: Learning Rate, Batch Size, Number of Layers, Dropout Rate, and Epochs. Learning Rate accuracy increases from 0.001 to 0.02 and peaks at 96.5%. This suggests that a greater learning rate improves the model to a point beyond which it may not improve. As batch size increases, accuracy improves, reaching 96.0% at 128. This shows that bigger batch sizes improve gradient estimations and performance. The number of Layers research shows that accuracy increases from 2 to 5 layers, reaching 95.0%. This shows the model can capture complicated patterns with deeper structures. Higher Dropout Rate numbers decrease accuracy, showing that a high dropout might hamper performance. At 95% accuracy, 0.1 to 0.2 seems to be the best range. Epochs consistently improve accuracy with training time, reaching 95.0% after 40. This suggests more extended training improves model learning and generalization. This Figure shows how tuning these hyperparameters affects RMGACNet model performance, helping academics and practitioners optimize model settings.

**Fig 13:**
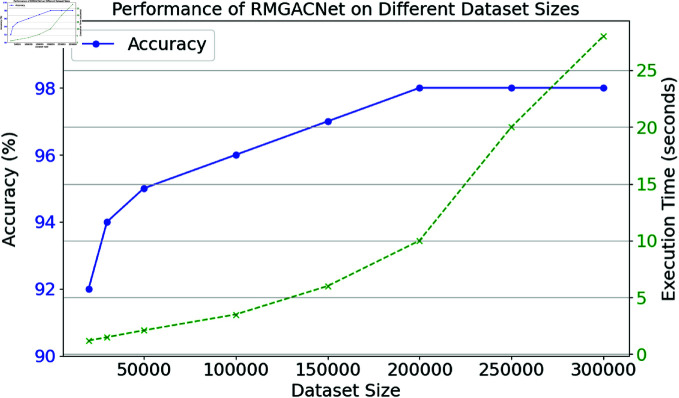
RMGACNet scalability and execution analysis with different dataset size.

Fig 13 shows the RMGACNet model’s performance across dataset sizes. The left y-axis shows accuracy percentages and the correct execution time in seconds. From 150,000 to 300,000 samples, the RMGACNet model’s accuracy steadily improves, stabilizing at 98%. The model uses more enormous datasets to improve its performance, resulting in excellent accuracy. However, execution time increases with increasing dataset sizes due to computing loads. In particular, execution time increases from 1.2 seconds for 20,000 samples to 28 seconds for 300,000 samples. The RMGACNet model, as seen in the image, allows for extending processing speeds without sacrificing accuracy. An adequate amount of computing power allows RMGACNet to operate in real time. When processing vast amounts of data, RMGACNet is a good option since it is more accurate and takes less time to calculate than earlier performance tests.

**Fig 14:**
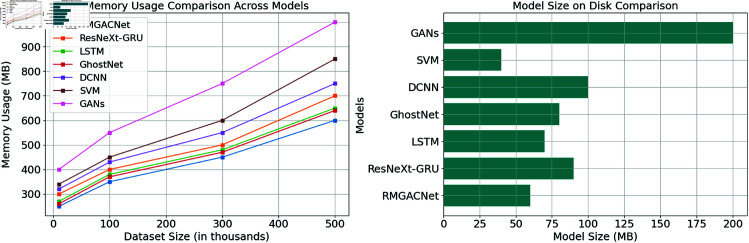
Memory usage and model size comparison across various models.

The memory usage and model size of RMGACNet, ResNeXt-GRU, LSTM, GhostNet, DCNN, SVM, and GANs are compared in Fig 14. The left figure illustrates the impact of dataset size on each model’s computational performance and memory usage. Even with larger datasets, RMGACNet consumes less memory than comparable models. The memory efficacy of RMGACNet may facilitate the processing of large datasets. Due to their substantial memory requirements, GANs and SVMs are not recommended for real-time applications. The model disk space is depicted in the right photo. RMGACNet consumes significantly less disk capacity than lesser variants. Due to its minimal memory and storage requirements, RMGACNet is an optimal choice for low-processing applications. GANs and SVM are effective but space-intensive, which may pose a challenge in real-world applications with restricted storage. RMGACNet is also demonstrated in Fig 14 to be an optimal choice for resource-intensive, large-scale fraud detection applications due to its storage and computational efficiency.

### Analysis of performance improvement

Compared to existing models, RMGACNet’s 2% to 7% performance improvement results from multiple, carefully integrated components, each addressing critical challenges in financial fraud detection. RMGACNet optimizes model performance via sophisticated regularisation and hyperparameter tweaking to exploit transaction data’s temporal and relational properties.

- Fraudulent activity usually features unusual timing or transaction frequency spikes. Sequence-processing constraints hinder standard models from capturing these details. RMGACNet solves this with BiLSTM layers that assess transactions both ways. Using this bidirectional method, the model learns from past and future transaction sequence data. RMGACNet’s BiLSTM layer improves memory and accuracy by detecting slight but significant time-based irregularities.
- Financial transactions sometimes include network structures like account links, which may reveal fraud tendencies with several transactions. RMGACNet learns node embeddings that represent transaction structural linkages and relational patterns using GraphSAGE. L2 regularization reduces overfitting to tiny training data variations by penalizing high-weight values. Financial data with class imbalance and high-dimensional noise need GraphSAGE embeddings to generalize across new data. This technique ensures this. Knowledge of structural data improves RMGACNet’s accuracy and AUC-PR.
- Capsule Network Hierarchical Pattern Recognition: Fraud detection requires detecting complex relationships like related accounts and repeating behavioural patterns. Standard neural networks handle neurons independently, whereas capsule networks better capture spatial and structural hierarchy. It gathers group-related data to help the RMGACNet model discover fraud trends, such as unusually high transaction sequences across associated accounts. This skill improves model specificity and reduces false positives by detecting fraud in datasets with distinct feature spaces.
- Attention-based selective focus: Only a few high-dimensional financial dataset properties assist in identifying fraud. RMGACNet’s attention method emphasizes fraud-related qualities and downplays irrelevant data. The attention technique reduces noise and improves categorization by focusing on transaction amount, frequency, and account connection patterns. By removing unnecessary data, our targeted technique enhances RMGACNet’s accuracy and F1 score.
- SHO hyperparameter tuning efficiency: Complex models like RMGACNet need hyperparameter modification for optimal performance. Traditional tuning methods are computationally demanding and may not discover the best configuration. Imitating hyena-hunting groups, SHO discovers an ideal set faster by exploring several combinations. Due to this adjustment, RMGACNet works effectively without overfitting by increasing its learning rate, dropout rate, batch size, and other parameters. Model stability and dataset consistency depend on this phase.
- SMOTE balances fraud detection datasets before training, helping RMGACNet perform better. It uses synthetic minority class samples to educate the model from fraudulent and non-fraudulent examples, improving generalizability and recall. By training RMGACNet on a balanced dataset, SMOTE avoids bias toward non-fraudulent transactions. Fraud detection requires better recall and specificity without overfitting to the majority class.

These components are combined to improve RMGACNet’s ability to identify fraudulent transactions. A model that surpasses current methods and adjusts to trends in financial fraud is created by combining attention-based feature selection with structural learning, hierarchical feature recognition, and temporal analysis. This model is then enhanced by hyperparameter tuning. RMGACNet considers the relational structure, class imbalance, and time dependence to improve performance metrics and optimizes the model. The revolutionary algorithms of RMGACNet’s fraud detection show how useful it may be in the banking sector.

### Limitations of RMGACNet

While RMGACNet’s recall and accuracy are crucial for fraud detection, the network has flaws. The computer resources required to evaluate massive datasets impact the model’s performance, which could hinder the scalability of real-time applications. In addition, as the model is hyperparameter sensitive, extra care must be used during implementation for optimal results. When RMGACNet is implemented, problems arise that require more study and enhancement.

## Conclusion

This research presents a new approach to identifying financial fraud using state-of-the-art deep learning algorithms. RMGACNet improves the efficacy of fraud detection systems by optimizing classification, feature extraction, and bidirectional long-short-term memory (BiLSTM) networks. To determine its effectiveness, we have extensively tested RMGACNet on the IEEE-CIS dataset and the European Cardholder (ECH) transactions dataset. The suggested solution outperforms prior approaches to financial fraud detection by using state-of-the-art deep-learning algorithms. RMGACNet deftly detects complex patterns and correlations in transaction data to identify fraudulent activity reliably. This approach ensures that it prioritizes the most critical aspects to improve the model’s prediction performance. Regarding accuracy, F1 score, and area under the ROC curve, RMGACNet often outperforms top approaches, demonstrating its capability to distinguish between legitimate and fraudulent transactions. Notwithstanding RMGACNet’s scalability and efficiency, which are appropriate for practical applications, it is vital to understand the ethical implications. The model must be closely monitored before being used in real-world scenarios to reduce the impact of biases caused by improperly selected analytical tools or biased training data.

Future studies should emphasize addressing ethical problems, ensuring openness, and promoting justice in fraud detection. By providing a comprehensive solution for detecting and mitigating fraud, RMGACNet has the potential to raise security standards in the financial industry. The model’s ability to analyze massive amounts of transaction data in real-time makes it useful for businesses aiming to reduce fraud risk. Even though it demonstrates a significant advancement in fraud detection technology, further study is needed to comprehend the ethical implications of RMGACNet and enhance its performance in various financial contexts.
